# A Case of Elephantiasic Pretibial Myxedema Successfully Treated With Intralesional Triamcinolone Acetate

**DOI:** 10.7759/cureus.29234

**Published:** 2022-09-16

**Authors:** Deep Hathi, Soumik Goswami, Nilanjan Sengupta, Arjun Baidya, Prashant M Gaikwad, Niladri Das, Silima S Tarenia, Rajul Oswal

**Affiliations:** 1 Endocrinology, Nil Ratan Sircar Medical College, Kolkata, IND; 2 Endocrinology and Diabetes, Nil Ratan Sircar Medical College, Kolkata, IND; 3 Endocrinology, Diabetes and Metabolism, Nil Ratan Sircar Medical College, Kolkata, IND; 4 Radiology, Shri Vinoba Bhave Civil Hospital, Silvassa, IND

**Keywords:** triamcinolone acetate, glucocorticoids, pretibial myxedema, graves dermopathy, graves disease

## Abstract

Graves' dermopathy is one of the extra-thyroidal manifestations of Graves’ disease (GD) and is characterized by the accumulation of glycosaminoglycans in the reticular dermis. In the majority of cases, pretibial myxedema is self-limiting but, in some cases, it can lead to structural and functional damage. Topical steroids with occlusive dressing remain the conventional treatment, but intralesional steroids have shown promising results. We hereby present a case of pretibial myxedema treated successfully with intralesional triamcinolone acetate.

## Introduction

Graves' dermopathy, which is one of the extra-thyroidal manifestations of Graves’ disease (GD), is caused due to local autoimmune response of the connective tissue. It is probably caused by antibodies against thyroid-stimulating hormone (TSH) receptors [1]. The prevalence of Graves’ dermopathy is estimated to be 0.5% -4% amongst patients with GD and is likely to be associated with severe Graves’ ophthalmopathy (GO) (15%) [2]. Even though the exact pathogenesis is not clear, pretibial myxedema can arise due to immunological factors, and mechanical factors like trauma and prolonged standing [2].

Pretibial myxedema is usually diagnosed clinically in the association of GO, goiter, and symptoms of hyperthyroidism. However, in certain cases, a biopsy is a must for confirmation of diagnosis [3]. Histologically, on haemotoxyllin and eosin staining (H&E), the tissue shows wide spacing between collagen bundles on alcian blue staining; it shows the accumulation of abundant mucopolysaccharides acid between collagen bundles of the dermis. The presence of activated fibroblasts seems to be the major pathogenesis of dermopathy [1,3].

Most cases of dermopathy are self-limiting in nature. it may be rarely serious enough to present with cosmetic issues. Traditionally, the application of occlusive corticosteroid over the lesion has been reported as successful therapy in past, however, with larger lesions, the clinical response decreases [1]. Recent use of intralesional corticosteroid has demonstrated better response in a shorter duration in large lesions, and we report one such case [4-6].

## Case presentation

**Figure 1:**
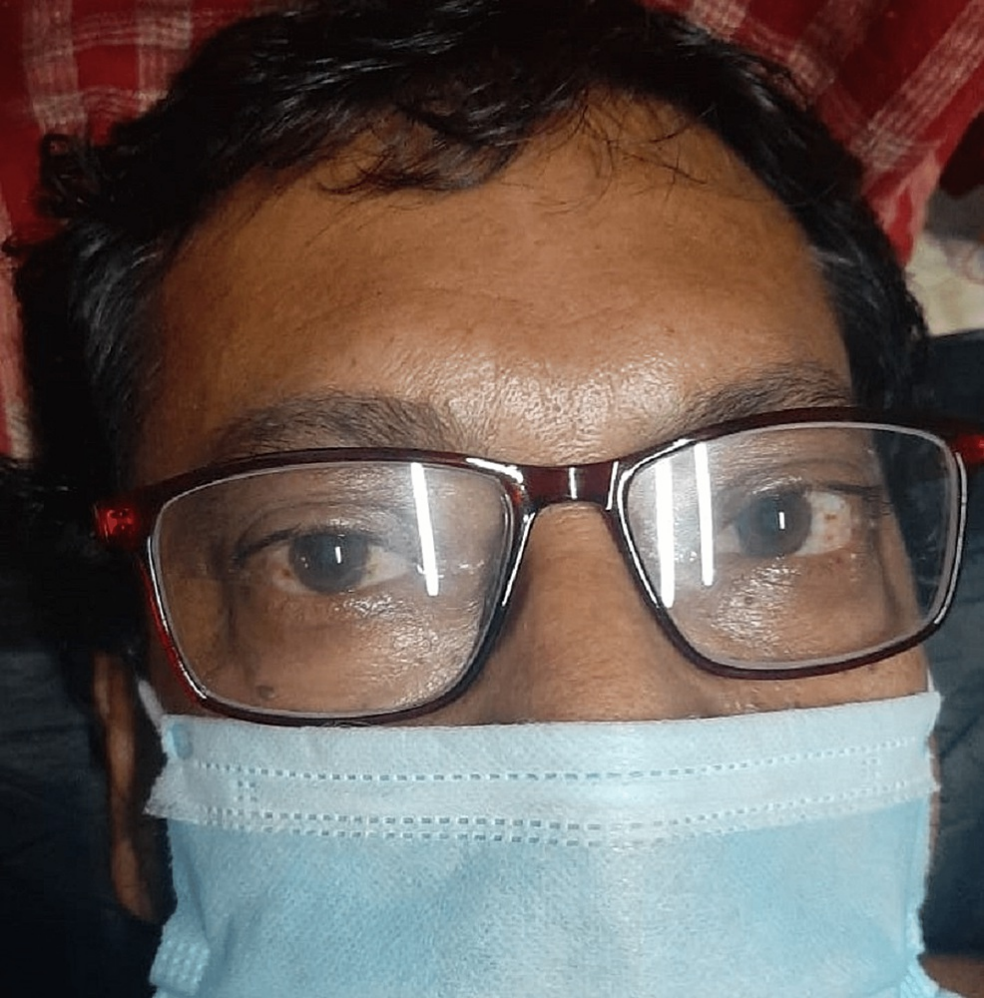
Graves' ophthalmopathy

**Figure 2:**
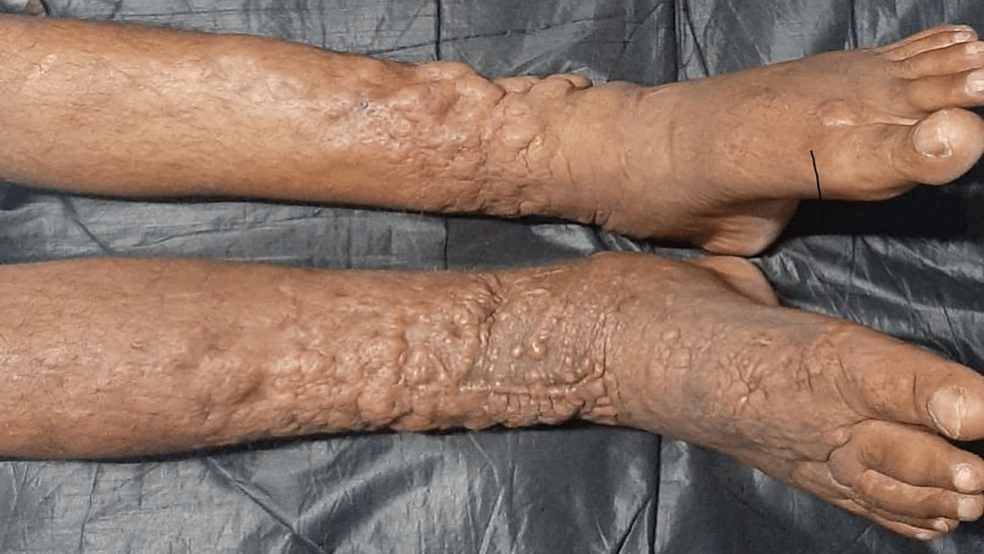
Multiple plaques and 'orange peel’ on the anterior surface of both the legs, ankles, and feet

A 53-year-old male patient, diagnosed with GD since 2016, presented with elephantiasic myxedema involving both lower limbs. The patient had bilateral exophthalmos with a clinical activity score (CAS) of 0/7 bilaterally (Figure 1). The patient was on tablet carbimazole 20 mg once daily with good compliance to therapy and was clinically and biochemically euthyroid. On clinical examination, there was non-pitting edema associated with nodules and yellowish-brown plaques giving rise to an elephantiasiform pattern on both the lower limbs involving the foot, ankle, and legs (Figure 2).

**Figure 3:**
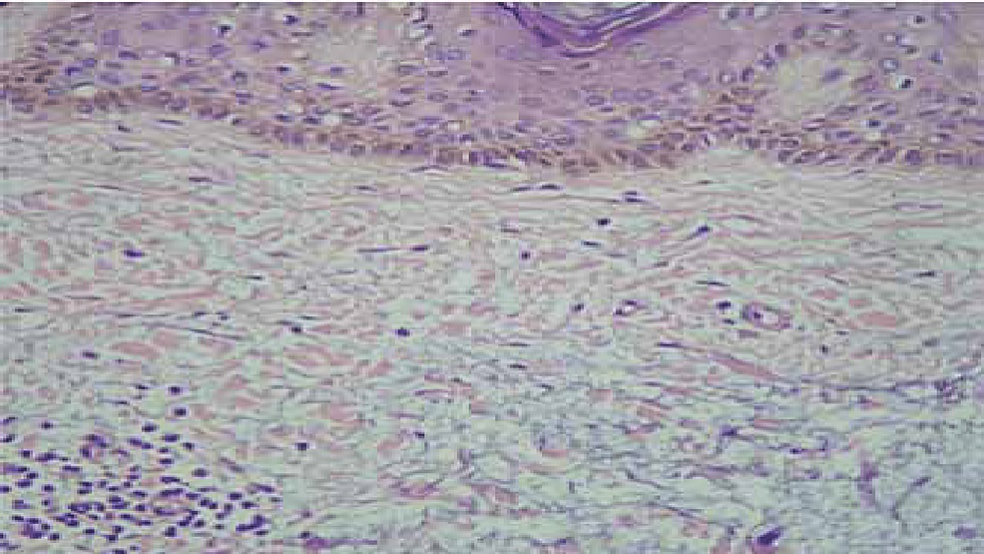
Histopathological examination (H&E, x100); accumulation of mucin in the reticular dermis causing separation of collagen bundles

On histopathological examination, the epidermis showed evidence of hyperkeratosis along with deposition of mucin within the collagen bundles in the reticular dermis confirming the diagnosis of pretibial myxedema (Figure 3).

**Figure 4:**
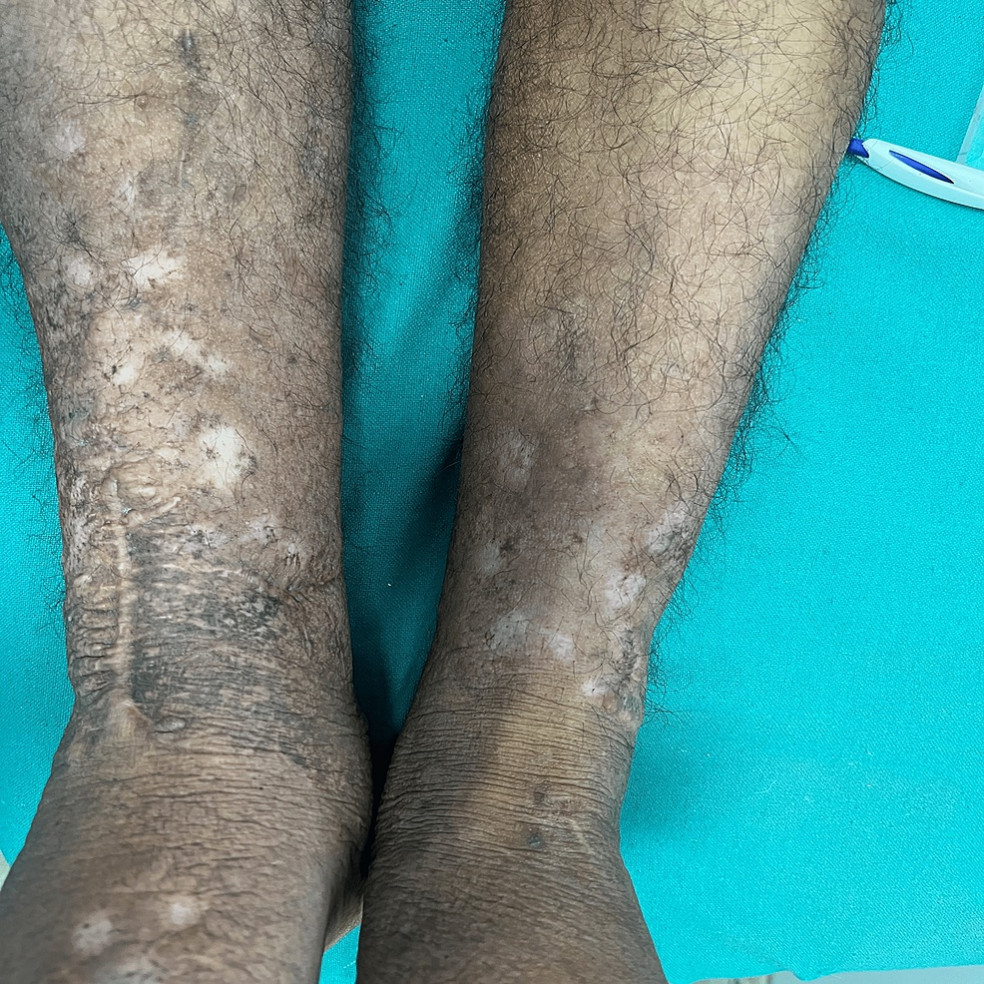
Recovery after four months of therapy with intralesional steroids

The patient was concerned about the unsightly look of his lesion and had difficulty using footwear. Keeping in mind the patient’s desire to improve his appearance, therapy with intralesional triamcinolone was planned. Triamcinolone 20 mg (10 mg/ml) was applied without dilution to multiple points under aseptic conditions - 0.1 ml per point via a 2 ml syringe with a 25 G x 0.5 inch needle into the dermis. The injection was repeated monthly and after four injections, a satisfactory response was achieved (Figure 4). The patient received his last injection six months back and there has been no recurrence.

## Discussion

GD is an autoimmune disorder characterized by autoantibodies against the TSH receptor. It is the leading cause of hyperthyroidism, predominantly affecting females with an overall incidence of 15 to 50 per 100,00 people a year. Extra-thyroidal manifestations of GD in order of frequency include exophthalmos (30%), pretibial myxedema (4%), and acropathy (less than 1%) [2]. 

Pretibial myxedema has a variable clinical presentation which can vary from non-depressible edema, which is the most common presentation to polypoid lesions, plaques, nodules, and features similar to elephantiasis. As the name suggests, the most commonly reported site is the pretibial region but it can present anywhere over the skin including the head, trunk, and limbs. The lesions are typically asymptomatic but can rarely be pruritic or painful. The color of the lesion varies and they may look like “orange peel” due to prominent hair follicles [1-3].

The aim of therapy in pretibial myxedema is to reduce the levels of hyaluronic acid by fibroblasts. To date, the various treatment options that were used are topical steroids with an occlusive dressing, systemic corticosteroids, pentoxifylline, plasmapheresis, immunotherapy, and surgical excision with variable success rates [1].

To achieve remission, topical steroids need to be applied with occlusive dressing daily at bedtime for 4-6 weeks in order. In contrast, intralesional corticosteroids are given as monthly injections with a faster rate of remission and it is less laborious for patients. The adverse effects reported with the use of intralesional steroids include hyperpigmentation, atrophy, and irregularities on the surface of the skin [1].

## Conclusions

Patients with severe pretibial myxedema can presents with cosmetic and functional impairment; treating such patients with modalities that can result in early recovery can reduce the unsightliness of lesions in patients. Our patient with elephantiasic pretibial myxedema showed excellent response to intralesional triamcinolone acetate, demonstrating the utility of the said treatment in this condition.
